# Is consumer behaviour towards footwear predisposing for lower extremity injuries in runners and walkers? A prospective study

**DOI:** 10.1186/s13047-019-0354-x

**Published:** 2019-08-17

**Authors:** Tine Marieke Willems, Roel De Ridder, Philip Roosen

**Affiliations:** Department of Rehabilitation Sciences, Ghent University, Ghent University Hospital, Corneel Heymanslaan 10, 3B3, 9000 Ghent, Belgium

**Keywords:** Runner, Walker, Risk factor, Injury, Footwear

## Abstract

**Background:**

Runners and walkers often suffer from lower extremity injuries. Little is known about the relationship between their consumer behaviour towards footwear and the development of those injuries. Therefore, the aim of this study was to investigate if consumer behaviour towards footwear is a risk factor for lower extremity injuries.

**Methods:**

A prospective cohort study was set-up in leisure-time walkers and runners. Potential risk factors in consumer behaviour were obtained by means of a baseline questionnaire related to the acquisition of current walking or running shoes. Information on injuries sustained during a 24 week period after the baseline questionnaire was obtained in 104 runners and 104 walkers using a 2-weekly questionnaire. Binary logistic regression analysis was used to identify risk factors for lower extremity injuries in the consumer behaviour.

**Results:**

Forty- nine (24%) subjects suffered a self-reported lower extremity injury. 35 injuries occurred in runners and 14 among walkers.

Undergoing a gait analysis before buying shoes was associated with an increased occurrence of lower extremity injuries (odds ratio (OR) 4.76). A protective factor was caring a lot about the right fitting of the shoes (OR 0.11).

**Conclusion:**

Runners and walkers should pay attention to the correct size when buying footwear to diminish the risk of lower extremity injury. Buying footwear after a gait analysis increased the risk of a lower extremity injury in runners and walkers, however, this might be associated with the increased risk that was already present because of previous injury.

**Trial registration:**

This trial was not registered since this was an observational study and no intervention took place.

**Electronic supplementary material:**

The online version of this article (10.1186/s13047-019-0354-x) contains supplementary material, which is available to authorized users.

## Introduction

Running is one of the most popular and accessible physical activities. For whom running puts too high demands on the cardiovascular or musculoskeletal system, walking is a good alternative with the same positive health benefits [1]. The simplicity of walking, associated with little cost, makes it economically accessible and thus one of the best ways to achieve recommended daily amounts of physical activity by the World Health Organization (WHO) [2]. Many countries added these recommendations in their own guidelines, which resulted in a small increase in the participation of walking [3]. However, running and walking also generate a non-trivial amount of running and walking related injuries. The reported incidence of running related injuries varies between 19 and 79% [4]. Walking injuries occur less frequently and the incidence rate is almost half of that of running [5]. The last 2 decades, many studies have focused on identifying risk factor for running related injuries. Numerous articles focused on the association between running related injuries and personal factors as sex, age, BMI and foot posture [6]. Additionally, there are also plenty investigating training related factors as distance, frequency, training intensity [6]. Also footwear related risk factors have been given attention, however, knowledge about footwear related risk factors is still limited. A recent systematic review showed that having used orthotics/inserts and changing shoes more frequently was associated with an increased risk of running injuries [6]. In other sport disciplines, it has been shown that sport shoes that fit well are protective against sports injuries [7, 8].

When looking for a new pair of running or walking shoes, the customer is overwhelmed by the possible choices. Since the introduction of the “modern” running shoe in the 1960s and the running boom in the 1970, the running shoe industry has steadily grown into a huge market [9]. Advertising campaigns all tell you that they sell the best footwear. Commitments of better stability and motion control, lower impact forces next to less fatigue, increasing speed, distance and performance are legion. Protection against injury is one of the most often used arguments by shoes manufacturers to justify sophisticated technological features. Notwithstanding those promises, still many running and walking related injuries occur and the injury incidence has not changed noticeably over the last few decades [10].

When buying footwear, every customer relies on certain criteria to make a choice [11]. Additionally, the customer is influenced by several other factors such as brand, product characteristics, quality and price [12, 13]. The basic decisions imply those decisions that a runner or a walker makes before he gets to the store, including the place of acquisition, whether or not undergoing a gait analysis, the price, a second-hand buy, the reason for acquisition, the influence of advice of others and impulsiveness. The influencing factors contain those factors that lead the consumer to choose that specific pair of shoes in the end, e.g. colour, model, material, presence of specific properties and price. Consumer behaviour is heavily motivated by advertising. In literature, it has been shown that there is no correlation between the price of the sport shoes and the quality, between the price and the comfort and between the price and the onset of stress fractures [14, 15]. But on the other hand, deceptive marketing of athletic footwear leads to a decreased caution and care on the part of the runner, potentially increasing injury risk [16].

At the moment, our capacity to prevent running and walking injuries is limited; training advise and footwear prescription form the mainstays [17]. Although that several footwear shops offer a gait analysis as part of a comprehensive in-store fitting service, Richards et al. showed that the current practice of prescribing running shoes adapted to the gait pattern is not evidence-based [18]. To be able to prevent injuries, prospective studies are needed which identify possible risk factors.

Since there is limited evidence of the effect of prescribed shoes on the occurrence of injuries and, to our knowledge, no studies have been performed on the relationship between consumer behaviour and the occurrence, we set out to do a prospective study to investigate if prescribed footwear and other consumer behaviour in runners and walkers was associated with running/walking injuries.

## Methods

### Subjects

Leisure-time runners and walkers were recruited for this prospective study via advertisements on local running and walking events. A total of 280 walkers and 300 runners initially agreed to participate in the study and completed the baseline questionnaire. Fifty-two (52) walkers and 93 runners were excluded from the study because they did not fulfil all the following in- and exclusion criteria: 1) walk or run at least 10 km per week, 2) no complaints at the lower extremities in the last 2 weeks, 3) no surgery at the lower extremities in the last 3 years, 4) no systemic disease, cardiac problems or diabetes. Participants had to maintain a minimal distance of ten kilometres per week, for a period of 24 weeks and needed to wear one and the same pair of shoes during their walking/running activities during this period. One hundred and twenty-four (124) walkers and 103 runners who did not complete all follow-up questionnaires or changed shoes during the investigation were excluded. Consequently, only the data of the remaining 104 walkers (53 men and 51 women) and 104 runners (57 men and 47 women) of 18 years and older were taken into account.

The study was approved by the Ghent University Hospital Ethical Committee and all participants signed an Informed Consent.

### Consumer behaviour questionnaire

All participants completed the baseline questionnaire measuring motives for purchasing their current running or walking shoes via an online survey system. The questionnaire was developed by the authors and included a question for every criterion that influences a possible buyer to perform a purchase hereby comprising 24 questions measuring basic decisions and influencing factors [11]. The *basic decision* survey included multiple choice questions including the place of acquisition, whether or not undergoing a gait analysis before buying (prescribed) walking/running shoes, the price, a second-hand buy, the reason for acquisition, the influence of advice of others and impulsiveness (Additional file 1). To evaluate the *influencing factors* participants indicated on a 5-point Likert scale (from ‘not at all’ to ‘very much’) to which degree the following factor influenced the purchase: colour, model, material, closure mechanism, presence of specific properties, price, quality, price quality ratio, sales and discounts, brand, fashion, advertisement, comfort, necessity, sport specificity, right fitting, technology and store service. The questionnaire was pilot-tested on a group of 574 athletes before this investigation and resulted in a small adaptation to the possible answers for ‘Reason for acquisition’ based on answers of the subjects and on expert opinion.

### Outcome measure

The outcome measure for this study was a self-reported running or walking injury occurring within 24 weeks after completing the baseline questionnaire. Injury occurrence was captured by means of an online injury questionnaire, which the subjects completed every 2 weeks, for a period of 24 weeks. Questions concerned the location of injury, pain intensity, complaints and consequences. The used injury definition was elaborated on the injury definition of Macera et al. [19] and Wen et al. [20]. A running or walking injury was defined as a self-reported “injury on muscles, joints, tendons and/or bones of the lower extremities (hip, groin, thigh, knee, lower leg, ankle, foot, and toe) that the participant attributed to running or walking.” The problem had to be severe enough to cause a reduction in the distance, speed, duration or frequency of running or walking or treatment of the injury was carried out (e.g. use of medication, visit to a health professional).

### Statistical data analysis

The subjects were divided into two groups: those who developed a running or walking injury throughout the monitoring period of 24 weeks (injured group) and those who did not (uninjured group).

Using running and walking injuries as the dependent variable, univariate logistic regression analyses were performed on the basic decision and influencing factors of the consumer behaviour for each factor independently. Next, a multivariable model was built using a stepwise method. Variables with a *p* value ≤0.2 on the Wald test in the univariate analysis were included in the multivariate model in a first step. Non-significant variables in the multivariable model were eliminated using *p* > 0.1. In a next step, variables that were not significant in the univariate analysis were added to the multivariate model obtained at the end of the previous step. *P* ≤ 0.1 was used to decide whether to add the variables or not.

Calibration of the logistic model was assessed using the Hosmer-Lemeshow goodness-of-fit test, and discrimination was assessed using the area under the receiver operating characteristic (ROC) curve to evaluate how well the model distinguished subjects who were injured from those who were not injured.

All statistical analysis were performed using IBM SPSS version 25 .

## Results

The baseline characteristics of the study population are represented in Table 1. The mean age of the included runners and walkers was 50 years. Ninety-eight participants were female (47%).

**Table 1:** Baseline characteristics of runners and walkers and their shoes (*n* = 208)

Characteristic	N (%)	Mean (SD)
Age (years)		49.97 (11.6)
Sex (female)	98 (47.1%)	
BMI (kg/m^2^)		21.84 (2.5)
Level of running/walking		
Recreational	199 (95.7%)	
Competitive	9 (4.3%)	
Professional	0	
Place of acquisition		
Sports shop	165 (79.3%)	
Shoe shop	24 (11.5%)	
Shop not specialized in selling shoes	7 (3.4%)	
Via internet	2 (1.0%)	
Via sports club	1 (0.5%)	
Other	9 (4.3%)	
Undergoing a gait analysis and buying prescribed shoes adapted to the results		
No gait analysis, no prescribed shoes	133 (63.9%)	
No gait analysis, prescribed shoes	6 (2.9%)	
Gait analysis, no prescribed shoes	12 (5.8%)	
Gait analysis, prescribed shoes	57 (27.4%)	
Second-hand buy	0 (0%)	
Average price of shoes (euro)		125.75 (43.0)
Influenced by advice of others	134 (64.4%)	

During the 24 weeks follow-up, 49 (24%) of the participants suffered one or more injuries of which 35 injuries occurred in runners and 14 among walkers. Twenty-eight injuries occurred in female participants (28%) while 21 injuries in male participants (19%). The lower leg and knee were the most common reported injury site (both 24.5%), followed by foot (18.4%) and ankle injuries (14.3%) (Table 2).

**Table 2:** Location of injuries

Location	N (%)
Foot	9 (18.4%)
Ankle	7 (14.3%)
Lower leg	12 (24.5%)
Knee	12 (24.5%)
Upper leg	4 (8.2%)
Hip	5 (10.2%)
Total	49

Results of the univariate analyses showed that 5 of the 24 potential risk factors measured at baseline were significantly associated with running/walking injuries (*p* < 0.05) (Table 3). Four of them increased the risk, namely undergoing a gait analysis, being influenced by advice of others, not caring about the model or the closure mechanism. Caring about right fitting decreased the risk of a running/walking injury.

**Table 3:** Risk factors for walking/running injuries

Variable	Univariate analysis		Multivariable analysis	
	Odds Ratio (95%CI)	*p*-value	Odds Ratio, (95%CI)	*p*-value
Basic decisions				
Place of acquisition		0.578		
Gait analysis before buying (prescribed) shoes (having no gait analysis and not buying prescribed shoes is the reference)				
Gait analysis and prescribed shoes	4.65 (2.25;9.58)	**< 0.001**	4.76 (2.26;10.02)	**< 0.001**
No gait analysis but prescribed shoes	3.19 (0.55;18.73)	0.198	2.28 (0.27;18.99)	0.447
Gait analysis but no prescribed shoes	4.56 (1.31;15.94)	**< 0.001**	4.75 (1.28;17.69)	**0.020**
Price		0.596		
Reason for acquisition		0.993		
Influenced by advice of others (no influence is the reference)	2.63 (1.22;5.64)	**0.013**		
Impulsiveness		0.268		
Influencing factors				
Colour		0.107		
Model (neutral is the reference)				
Not at all	4.62 (1.66;12.88)	**0.003**		
Not	2.91 (1.05;8.06)	**0.040**		
Much	0.84 (0.35;2.03)	0.689		
Very much	0.73 (0.22;2.43)	0.608		
Material		0.061		
Closure mechanism (neutral is the reference)				
Not at all	5.27 (1.71;16.27)	**0.004**		
Not	3.25 (1.09;9.66)	**0.035**		
Much	1.51 (0.64;3.53)	0.345		
Very much	1.15 (0.36;3.67)	0.818		
Presence of special properties		0.309		
Price		0.459		
Quality		0.252		
Price/quality ratio		0.208		
Sales and discount		0.962		
Brand		0.380		
Fashion		0.773		
Advertisement		0.275		
Comfort		0.939		
Necessity		0.663		
Sport specificity		0.118		
Right fitting (neutral is the reference)				
Much	0.11 (0.02;0.57)	**0.009**	0.11 (0.02;0.67)	**0.017**
Very much	0.30 (0.06;1.39)	0.123	0.29 (0.05;1.63)	0.160
Technology		0.343		
Store service		0.870		

Only for significant variables the odds ratios are shown. Only variables with a *p*-value > 0.2 in the univariate analysis were considered for the multivariable analysis. *P*-values < 0.05 are reflected in bold

The final multivariable logistic model after backward elimination is represented in Table 3. Undergoing a gait analysis and buying the prescribed or other shoes (OR 4.76; 95% Confidence Interval (CI): 2.26–10.02 and OR 4.75; 95% CI: 1.28–17.69 respectively) increased the risk of lower extremity injuries. Caring a lot about the right fitting (OR 0.11; 95% CI: 0.02–0.67) was a protective factor for the occurrence of those injuries.

The Hosmer–Lemeshow goodness-of-fit (*P* = 0.74) showed no lack of fit of the final model to the data (a large *P*-value indicates that there is not a large discrepancy between observed and expected injuries). The index of predictive discrimination for this model, namely the area under the.

ROC curve, was 0.74, reflecting fair ability of the model to discriminate between runners/walkers who do and do not have a running/walking injury.

## Discussion

In this cohort study, 34% of the runners and 13% of the walkers developed a lower extremity injury. The incidence rates in this study are comparable with previous study findings which also demonstrated that walkers are less susceptible to injuries than runners [5, 21]. In the current study there was no dominant injury region although knee and lower leg were the most common injured regions (each 24.5%). These findings coincide with the main running-related injury sites in the review of Lopes et al. [22].

This study has identified several risk factors for the occurrence of walking/running related injuries on the lower extremities in recreational walkers and runners. Caring about right fitting was shown to be a protective factor for lower extremity injuries. On the other hand, undergoing a gait analysis before buying shoes, being influenced by advice of others and not caring about the model or the closure mechanism increased the risk of walking/running injuries.

The main finding was that caring about buying the correct size decreased the risk of a lower extremity injury. The runners/walkers who cared about the correct size were 9 times less likely to develop injuries. Accordingly, studies in climbing showed that shoes that fit well are protective against sports injuries [7, 8]. It seems obvious that in shoes that are too big the shear forces will increase while shoes that are too small will put stress on the foot. Both scenarios would probably lead to increased load on the musculoskeletal system.

Univariate analysis showed that not caring for the model or the closure mechanism increased the risk of an injury. There was a strong correlation between carrying about the right fitting and carrying about the model and closure mechanism. This entails that those subjects that care about the correct size also care about the model and the closure mechanism. Because of the collinearity between those variables, only caring about the correct size was maintained in the final model. In general, making a well-considered choice seems to lower the risk of an injury. Taken together, we suggest that when buying a new pair of shoes, right fitting will provide the best basis for adequate adaptation to forces on the lower extremity during running/walking. Runners and walkers should pay special attention to the correct size when buying footwear to diminish the risk of lower extremity injury.

According to the results of this study, after undergoing a gait analysis the odds for a lower extremity injury increased almost 5 times. At first sight, this seems to be illogical since wearing prescribed footwear is supposed to lower the injury risk. However, other studies in military populations investigating the effectiveness of matching running shoes according to foot shape showed no influence on injury risk [23–25]. A meta-analysis that pooled results of 3 investigations showed little difference between the group with matching running shoes and the control groups in the injury rate (injuries per 1000 person-days) for either men (summary rate ratio = 0.97; 95% CI: 0.88, 1.06) or women (summary rate ratio = 0.97; 95% CI: 0.85, 1.08). When injury rates for specific types of running shoes were compared, there were no differences [26]. In addition, Van der Worp et al. recently showed in a systematic review that having used orthotics/inserts was associated with an increased risk of running injuries [6].

The explanation for this observation is not straightforward and we can only speculate about the involved mechanism. A first and probably key explanation might be that the population who underwent a gait analysis was predisposed to develop sports injuries because of previous injuries. Previous studies have described an injury history as the most predisposing factor for a new injury [19, 21, 27–29]. People who have an injury history are more likely to undergo a gait analysis, hoping not to develop any further injuries by procuring individually adapted shoes. Furthermore, the population which had undergone an analysis might consists of rather fervent runners or walkers. Van der Worp et al. showed that distance has an important influence on the risk of injury and therefore, risk might be increased just because of the level of exposure [6]. A second possible explanation might be that runners presume to have the perfect shoes with optimal protection against injuries after such a gait analysis. Consequently, they become unconsciously imprudent and take more risks while walking/running. In a previous study of Robbins and Waked a similar phenomenon was perceived [16]. Induced by advertisements, runners were highly confident that their sports shoes were of superior quality and as a consequence, they were less cautious when running and increased their risk of injury.

Although that we cannot prove that this injury history or incautiousness was the reason for the increased risk, we neither can demonstrate that the gait analysis was not performed well nor that the shoes were not selected properly. The way the gait analyses was performed may vary between subjects. Some subjects might have had a 2D/3D analysis while others might have undergone static and/or dynamic foot pressure measurements. The information such analysis offers is very variable and might be interpreted in a different way by the sellers. Next to that, it may also assist in awareness and understanding of previous injuries. So, perhaps the prescribed shoes did change the running/walking pattern to unload certain structures but simultaneously increased the load on other structures. However, the fact that there was a similar increase in the odds for injuries when subjects underwent a gait analysis but did not buy the prescribed shoes adapted to the result, lets us presume to believe that especially those with an increased risk because of an injury history undergo a gait analysis. Future research should however explore those hypotheses further and might give more insight in the articulation of the impact of gait analysis and the effect of it on the injury risk.

Being influenced by others during the acquisition of shoes also increased the risk of a lower extremity injury. Probably the same reasoning about reduced cautiousness applies as above, since there was a strong correlation between being influenced and undergoing a gait analysis. Almost half of those who indicated to be influenced during acquisition also underwent a gait analysis.

There are a lot of basic decisions and influencing factors which do not influence the risk of injuries of the lower extremity in walkers/runners. One of these is the price of the walking/running shoes, which confirms the previous findings of no association between the costs of the shoes and the development of stress fractures [14].

### Limitations

Individual email reminders were sent to participants who did not comply with injury reporting over the previous 2 weeks. However, participants that did not react to the email reminders, were excluded from the study. A possible selection bias could have occurred when an injury would have been the reason for dropping out. Next to that, given that the participants were asked to use their running/walking shoes during the entire study duration of 6 months, runners and walkers preferring to use more than one pair were excluded. Thus, the volunteers in this study may not represent all recreational runners and walkers.

Since no validated questionnaire to assess consumer behaviour could be found at the time of this investigation, the baseline questionnaire to measure consumer behaviour used in this investigation was not validated. Another limitation is that the questionnaire was not completed at the time point of the purchase itself. It is possible that the shoes were bought some time before the investigation which could have affected the reproduction of the consumer behaviour in the questionnaire.

Total sport exposure time was not measured in this investigation. Therefore, a more accurate time-to-event analysis could not be performed. In the current analysis we therefore rely on the assumption that sports exposure was similar in both the injured and non-injured groups.

## Conclusion

Caring for right fitting during the purchase of footwear is protective against developing injuries. Therefore, it is of utmost importance to buy appropriately sized footwear. Participants who had bought their footwear after a gait analysis had an increased risk of a lower extremity injury. This is possibly due to the fact that runners/walkers with a history of previous injuries are the ones who choose to undergo a gait analysis. People might think that after a gait analysis, they are protected against injuries but this seems not to be true. Therefore, runners or walkers who buy prescribed footwear after a gait analysis, should still be aware that this footwear does not prevent injuries from occurring.

## Additional file

Additional file 1: List of possible answers for basic decision questions concerning buying current running/walking shoes. (DOCX 19 kb)

## Data Availability

Please contact author for data requests.

## Abbreviations

OR: Odds ratio
CI: Confidence interval
ROC: Receiver operating characteristic
