# Supplementary material for: Is consumer behaviour towards footwear predisposing for lower extremity injuries in runners and walkers? A prospective study
Source: J Foot Ankle Res. 2019 Aug 17;12:43. doi: 10.1186/s13047-019-0354-x (PMC6697916; doi:10.1186/s13047-019-0354-x)
Supplement: Supplementary file 1 — List of possible answers for basic decision questions concerning buying current running/walking shoes. (DOCX 19 kb) [file 13047_2019_354_MOESM1_ESM.docx]

Additional file

Additional file 1: List of possible answers for basic decision questions concerning buying current running/walking shoes

1. **Place of acquisition**

- sports shop
- shoe shop
- shop not specialized in selling shoe (f.i. Aldi, Lidl, H&M)
- via internet
- via sports club
- other

1. **Undergoing a gait analysis**

- I bought my shoes without undergoing a gait analysis and did not buy shoes based on the results of a gait analysis
- I bought my shoes without undergoing a gait analysis, but the shoes were selected based on the results of a gait analysis
- I bought my shoe after undergoing a gait analysis, but I did not buy the selected shoes based on the results of the gait analysis
- I bought my shoes after undergoing a gait analysis and I bought the selected shoes based on the results of the gait analysis

1. **Price of your shoes:**

- € 0-20
- € 21-40
- € 41-60
- € 61-80
- € 81-120
- € 121-160
- € 161-200
- € 201-250
- > € 250

1. **Are your current shoes second-hand shoes?** (Yes/No)
2. **Reason for acquisition**

- Previous shoes were worn out or broken
- Previous shoes were dirty
- Previous shoes did not fit well
- I was bored with my previous pair of shoes
- New sport specific acquisition
- Because injury occurred wearing previous shoes

1. **Influence of advice of others: Did you buy your current shoes taking into account the advice of others (trainer, seller, relative, team members, physician, expert, …?** (Yes/No)
2. **Impulsiveness when buying shoes**

- Very impulsive
- Impulsive
- Thoughtful
- Very thoughtful
